# Extreme temperature impairs growth and productivity in a common tropical marine copepod

**DOI:** 10.1038/s41598-019-40996-7

**Published:** 2019-03-14

**Authors:** Nam X. Doan, Minh T. T. Vu, Hung Q. Pham, Mary S. Wisz, Torkel Gissel Nielsen, Khuong V. Dinh

**Affiliations:** 1 Cam Ranh Centre for Tropical Marine Research and Aquaculture, Institute of Aquaculture, Nha Trang University, No 2 Nguyen Dinh Chieu Street, Nha Trang City, Vietnam; 2 Present Address: World Maritime University, Fiskehamnsgatan 1, Malmö, Sweden; 3 National Institute of Aquatic Resources, Technical University of Denmark, Lyngby, Denmark

## Abstract

Shallow, tropical marine ecosystems provide essential ecosystem goods and services, but it is unknown how these ecosystems will respond to the increased exposure to the temperature extremes that are likely to become more common as climate change progresses. To address this issue, we tracked the fitness and productivity of a key zooplankton species, the copepod *Pseudodiaptomus annandalei*, acclimated at two temperatures (30 and 34 °C) over three generations. 30 °C is the mean temperature in the shallow water of the coastal regions in Southeast Asia, while 34 °C simulated a temperature extreme that occurs frequently during the summer period. For each generation, we measured the size at maturity and reproductive success of individuals. In all three generations, we found strong negative effects of warming on all measured fitness-related parameters, including prolonged development time, reduced size at maturity, smaller clutch sizes, lower hatching success, and reduced naupliar production. Our results suggest that *P*. *annandalei* are already exposed to temperatures that exceed their upper thermal optimum. Increased exposure to extreme temperatures may reduce the abundance of these tropical marine copepods, and thus reduce the availability of resources to higher trophic levels.

## Introduction

Temperature is one of the most important eco-physiological variables affecting the survival, abundance and performance of ectothermic organisms^1–3^. Increased sea surface temperature^4,5^ has become one of the major drivers of the change in structure and functioning of coastal marine ecosystems worldwide^6–9^. In tropical marine ecosystems, sea surface temperature increased more than 1 °C over the past 100 years and the rate of warming is expected to triple or quadruple by the end of the 21 century^4,10^. Shallow, coastal waters in the tropics are particularly prone to experiencing temperature extremes in a warming climate^11^. Such extreme temperatures are expected to negatively impact thermally specialised species occurring in these zones^12,13^ and many species already approach their upper thermal limits^14,15^.

Species can respond to warming by shifting distribution^16,17^, shifting phenology^18–20^ or changing morphological traits such as reducing body size^21,22^. A reduction of body size has been documented with warming temperatures in various taxa from marine phytoplankton^23^, zooplankton^21,24,25^, benthic invertebrates^26^, and fish^27^. This also aligns with the predictions of Bergmann’s rule, whereby tropical species tend to be smaller than related taxa at higher latitudes^27^. Warming has also been shown to impact fitness parameters in many taxa, either positively or negatively, depending on the taxa. For example, elevated temperature increased fitness over multiple generations in high latitude zooplankton^28^, but reduced the reproductive success of a coral reef damselfish *Acanthochromis polyacanthus*^29^. However, it remains relatively unknown how tropical coastal marine organisms, especially those at the base of the marine food web such as copepods, will be affected by extreme warming, particularly across generations.

In the tropical coastal ecosystems in the Indo-Pacific region the calanoid copepod *Pseudodiaptomus annandalei* is highly abundant^30,31^. They are also important grazers on small plankton^32^ and prey for fish larvae^31^. Any effects on fitness and productivity of this species are expected to have bottom-up controls that reduce food resources for small fish^31^ and top-down controls that reduce grazing rate on phytoplankton^33^, with ecological consequences. Here, we experimentally tested the effect of extreme temperature on the copepod *P*. *annandalei* by exposing them to the extreme temperature for three consecutive generations: F1, F2 and F3. The objective of this study is to assess the ecological consequences of the extreme temperature over the course of three generations. We documented how warming affects key fitness-related traits of copepods as the secondary producers including the development time, size at maturity of males and females, the clutch size, the hatching success and the naupliar production.

## Results

### Development of P. annandalei

**Figure 1:**
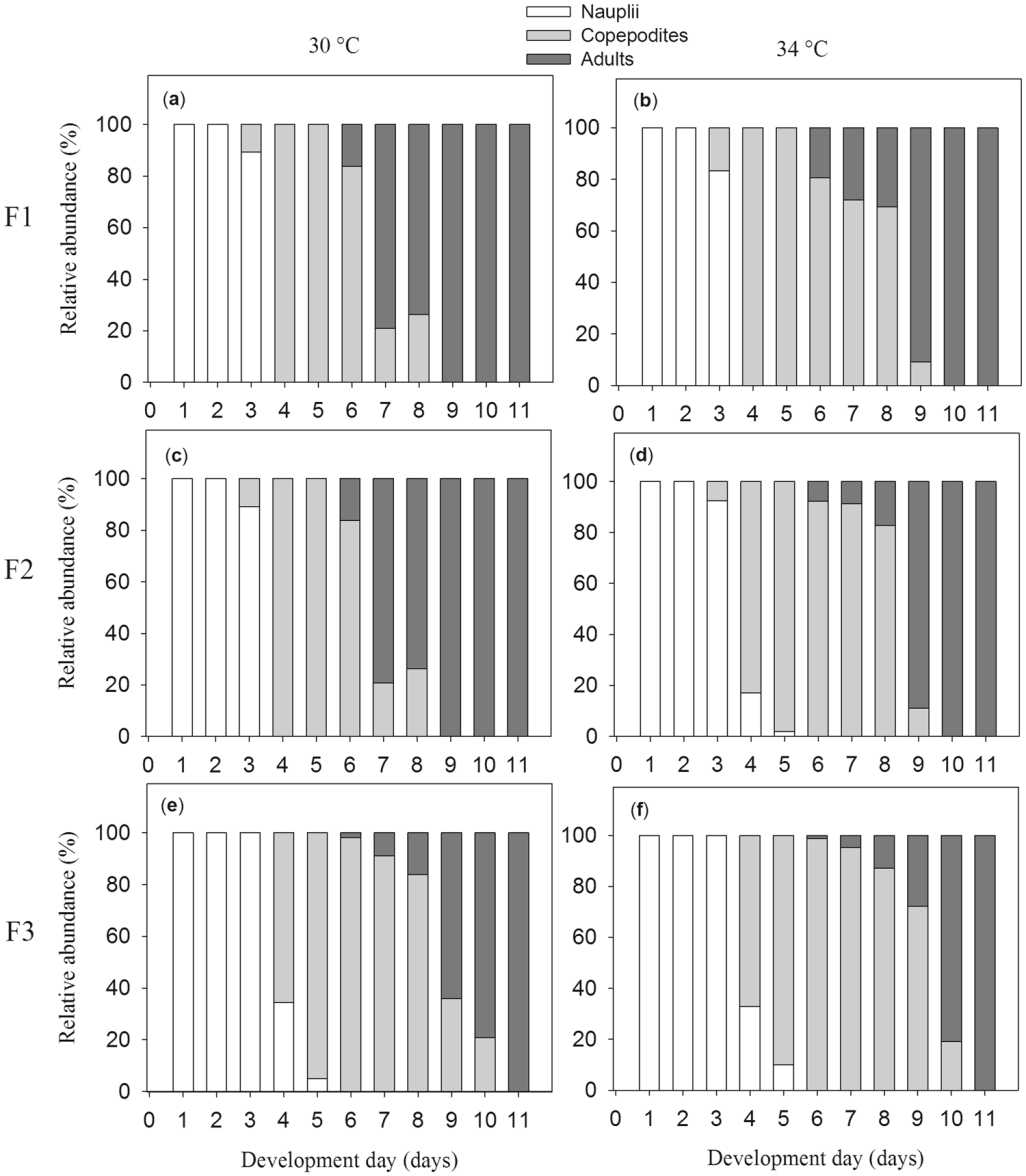
The development of *Pseudodiaptomus annandalei* in response to temperatures across three generations F1, F2 and F3. Data are the relative percentage of nauplii, copepodites and adults.

In the F1 and F2 generations, *P*. *annandalei* showed no difference in naupliar development time between 30 °C and 34 °C (Fig. 1a–d). The naupliar stages lasted from day 1 to day 3 after hatching. In contrast, the copepodite stages lasted approximately 2 days longer at 34 °C than at 30 °C (Fig. 1a–d). The adult stage started on day 7 at 30 °C and on day 9 at 34 °C (Fig. 1a–d). The nauplii development time of the F3 generation was similar to the F1 and F2 generations, but the copepodite stages lasted longer, and they reached the maturity on day 9 at 30 °C and day 10 at 34 °C (Fig. 1e,f).

### Size at maturity

**Figure 2:**
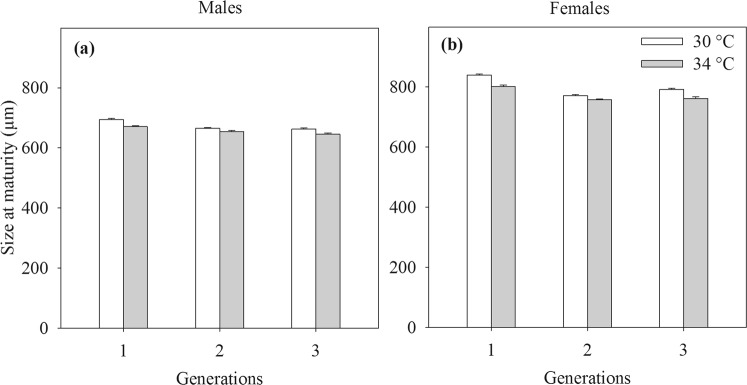
Size at maturity of males (**a**) and females (**b**) of the copepod *Pseudodiaptomus annandalei* as a function of temperature and generation. Data are means + 1 SE.

In both sexes, the exposure to extreme temperature resulted in smaller size at maturity (main effect Temperature). For males, the difference in size at maturity between 30 and 34 °C was detectable all three generations as the interaction of Temperature × Generation was not significant. Females showed a more pronounced size reduction in F1 than in F2 and F3 generations (interaction of Temperature × Generation, Table 1, Fig. 2). In both sexes, the size at maturity of F1 was larger than of F2 and F3 (for both males and females, F1 > F2, *P* < 0.001, F1 > F3, *P* < 0.001, Turkey posthoc tests). For males and females (at 34 °C), there was no difference between the two subsequent generations (Males: *P* = 0.99 at 30 °C, and *P* = 0.72 at 34 °C; and Females: *P* = 0.98 at 34 °C, Tukey posthoc test). At 30 °C, the size at maturity of F3 females was larger than of F2 females (*P* = 0.0015, Turkey posthoc test). Overall, the size at maturity of males and females of F1, F2 and F3 generations was smaller than of F0 generation (*P* values < 0.001, Tukey posthoc tests).

**Table 1:** The results of generalised mixed models, using bottle as a random effect, testing for the effects of the temperature on the size at maturity of males and females of the copepod *Pseudodiaptomus annandalei* across three generations.

Effects	Males			Females		
	df1,df2	F	P	df1,df2	F	P
Temperature	1, 540	32.45	**<0.001**	1, 500	65.48	**<0.001**
Generation	2, 539	35.29	**<0.001**	2, 500	137.17	**<0.001**
Temperature × Generation	2, 539	1.64	0.19	2, 500	5.82	0.0032

Significant *P* values (*P* < 0.05) are indicated in bold.

### Clutch size

**Figure 3:**
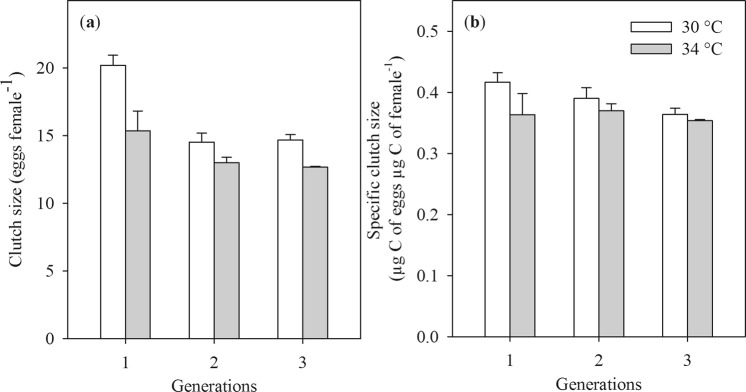
The clutch size (**a**) and specific clutch size (**b**) of the copepod *Pseudodiaptomus annandalei* as a function of the temperature and the generation. Data are means + 1 SE.

The number of eggs per clutch (two egg sacs) was lower in females reared at 34 °C than that of 30 °C (Table 2, Fig. 3). The clutch size was larger in the F1 generation than in F2 and F3, but there was no difference of clutch size between the two later generations (main effect Generation, and the interaction of Temperature × Generation, Table 1, Fig. 3). The effects of temperature and generation on the clutch size were still significant when the clutch size was normalized for the size of females (specific clutch size, Table 2, Fig. 3b). At 30 °C, the specific clutch size of females decreased after each generation (main effect of Generation, Table 2, Fig. 3b). The interaction between temperature and generation on specific clutch size was not significant (Table 2).

**Table 2:** The results of 5 responsive variables as a function of temperature for the copepod *Pseudodiaptomus annandalei* across three generations.

Effects	Clutch size			Specific clutch size			Hatching success			Nauplii production			Specific nauplii production		
	df1,df2	F	P	df1,df2	F	P	df1,df2	F	P	df1,df2	F	P	df1,df2	F	P
Temperature	1, 191	61.45	**<0.001**	1, 191	9.98	**0.0019**	1, 210	9.90	**0.0019**	1, 24	52.79	**<0.001**	1, 24	29.21	**<0.001**
Generation	2, 191	54.17	**<0.001**	2, 191	3.81	0.024	2, 210	0.02	0.99	2, 24	10.85	**<0.001**	2, 24	1.72	0.20
Temperature × Generation	2, 191	8.80	**<0.001**	2, 191	2.22	0.11	2, 210	0.02	0.99	2, 24	4.97	**0.016**	2, 24	2.17	0.14

The first three response variables (clutch size, specific clutch size, hatching success) were modelled with GLMMIX using bottle as a random effect. The remaining two response variables (nauplii production and specific nauplii production) were modelled using linear model. Significant *P* values (*P* < 0.05) are indicated in bold.

### Hatching success

**Figure 4:**
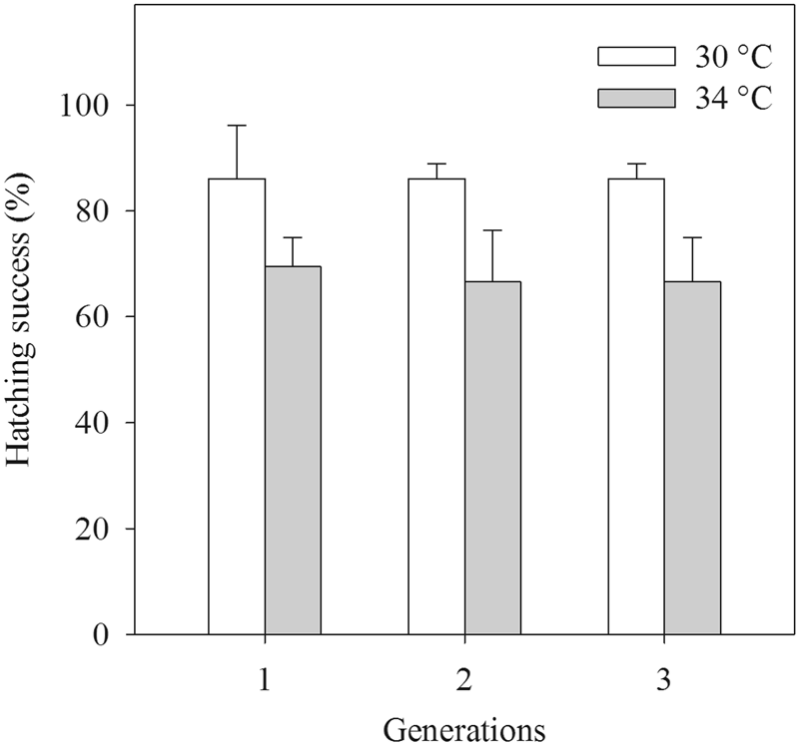
The hatching success of the copepod *Pseudodiaptomus annandalei* as a function of temperature and generation. Data are means + 1 SE.

The hatching success was 70% at 34 °C (thus approximately 20% lower than that at 30 °C) and this pattern was independent of generations (Table 2, Fig. 4).

### Naupliar production

**Figure 5:**
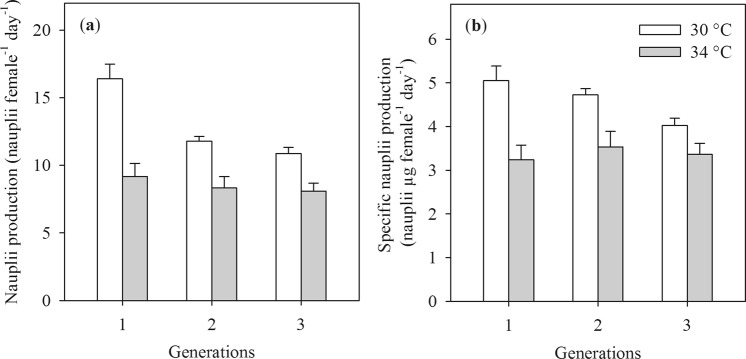
Nauplii production (**a**) and specific nauplii production (**b**) of the copepod *Pseudodiaptomus annandalei* as a function of the temperature and the generation. Data are means + 1 SE.

Nauplii and specific nauplii production were considerably lower at 34 °C than at 30 °C (main effect Temperature, Table 2, Fig. 5). The difference in nauplii production between temperatures was more pronounced in F1 generation than in the F2 and F3 generations (Temperature × Generation interaction, Table 2, Fig. 5a,b). Nauplii production of the F1 generation was higher than in the F2 and F3 generations while there was no difference between two later generations. The effect of generation and its interaction with temperature on nauplii production were lost after correcting nauplii production for the biomass of the females (Table 2, Fig. 5a,b).

## Discussion

In general higher temperature reduced all measured fitness-related traits of the tropical coastal copepod *P*. *annandalei* such as the size at maturity and reproductive success: clutch size, hatching success and nauplii production. A reduction in body size induced by warming is a widespread phenomenon in aquatic ecosystems^21–24,26,34^ but this is the first time this pattern has been documented for *P*. *annandalei*, a key zooplankton species in the tropical coastal environment. An accelerated development that outpaces growth rate in response to temperature increase is thought to explain the reduced size at maturity of marine copepod species^35,36^. However, this cannot explain the pattern in *P*. *annandalei* as the development time of this species was longer at 34 °C than at 30 °C. The higher, suboptimal temperature may result in a higher energy expenditure on basal metabolism (indicated by a higher respiration^37^). It may also intensify energy expenditure on additional biological functions such as the upregulation of heat shock proteins^38^, a universal mechanism used by many animals to cope with the thermal stress^39^ by diverting energy away from somatic growth and development. Furthermore, the energy from food prey may also be reduced under elevated temperature as the grazing rate of *P*. *annandalei* strongly decreased at 35 °C compared to 30 °C^33^.

Interestingly, the overall size at maturity of F1 was larger than those of F2 and F3 generations, irrespective of temperatures. Nauplii used to start F1 generation were hatched from wild collected females with a short-term acclimation period to the experimental temperatures. It was likely that the acclimation period of three days (approximately one-third of the generation time) may not fully eliminate the field conditions on F1 generation as size of F0 > control F1 > control F2. The culture conditions in the laboratory may also contribute to the changes in size at maturity of *P*. *annandalei* in the control treatment across generations. The control F3 females showed an increased size at maturity compared to control F2 females, indicating an adaptation to culture conditions other than temperature. However, this would not change our general finding that extreme temperature reduced the size of *P*. *annandalei* across three generations. The reduced size at maturity may have ecological consequences as adult size is positively associated with the grazing rate^40^, and reproductive output^24^. Our results supported this as they showed that smaller females exhibited lower nauplii production at 34 °C (see further below).

The clutch size was smaller at the temperature of 34 °C compared to 30 °C. Similar patterns have been observed in other copepod species e.g. *Pseudodiaptomus pelagicus*^41^ and *Tigriopus californicus*^42^. It has likewise been shown that the clutch size and egg production of marine copepods^43,44^ may be positively associated with the size of females. Although the smaller size of female *P*. *annandalei* at 34 °C may partly contribute to the smaller clutch size, the effect of extreme temperature on clutch size of *P*. *annandalei* was still significant after the clutch size was normalized for the biomass of females (specific clutch size). This suggests that egg formation and development may have been physiologically impaired by extreme temperature.

The hatching success of *P*. *annandalei* in the control temperature was high (80–90%), and was similar to that observed in previous studies^45,46^, while hatching success of *P*. *annandalei* was 70% at 34 °C. Previous work has shown that increased temperature can decrease hatching success^47^. For example, although *C*. *finmarchicus* hatching success was not affected at the range of the temperature from 6 to 19 °C, it was reduced to 25% at 22 °C and none of the eggs hatched at temperature of 25 °C^47^, possibly due to poor maternal provisioning^48^. Poor maternal provisioning may explain the lower hatching success in *P*. *annandalei* as they showed a reduced grazing rate at 34 °C compared to 30 °C^33^. Additionally, exposure to the extreme temperature of 34 °C may have negatively affected on the development of embryos, thereby contributing to the lower hatching success. Previous studies have shown a direct negative effect of extreme temperature on the embryonic survival of some spiders^49^ and marine fish^50^.

As a consequence of the smaller clutch size and lower hatching success, nauplii production was lower at 34 °C than at 30 °C. The reduced clutch size, hatching success and nauplii production at 34 °C were similar across all three generations and independent of generation, indicating no thermal acclimation and/or adaptation developed after three generations. Interestingly, the specific nauplii production of the control females decreased after each generation, similar to the pattern observed for specific clutch size. Some studies have shown that some transgenerational effects of e.g. antidepressants^51^ and cyanobacterial toxin^52^ on parental conditions may last at least three generations. This may be the case of *P*. *ananndalei* outside the laboratory.

While a number of studies have shown a rapid acclimation and/or adaptation of tropical marine species such as the coral damselfish *Acanthochromis polyacanthus*^53^ to global warming, this may not be the case for the tropical copepod *P*. *annandalei*. It is unlikely that this species could evolve the capacity to adapt to extreme temperature after only three generations. A previous study carried out in the shallow coastal waters of the Southeast Asian region showed that warming, together with other anthropogenic disturbance, promotes the dominance of opportunistic small copepod species such as *Oithona simplex*, *Hemicyclops* sp., *Pseudomacrochiron* sp. and *Microsetella norvegica* and a decrease in large-body copepod species such as *Pseudodiaptomus bowmani*, a congeneric species to *P*. *annandalei*^54^. While this pattern will be complicated by a number of other factors, particularly food quantity and quality, extreme warming may drastically change the structure, function and dynamics of coastal food webs as have been observed in a global biodiversity hotspot in the subtropical region^7^.

Copepods account for approximately 80% of the biomass of mesozooplankton and play a key role as major secondary producers, transferring energy from photosynthesis organisms to higher trophic levels^31,55^. Understanding the responses of tropical copepods such as *P*. *annandalei* to extreme warming is crucial for understanding the transfer of energy in coastal food webs^31^. Our study shows that extreme warming may negatively affect the secondary production by lowering copepod biomass and reproductive success. We found a reduction in *P*. *annandalei* reproductive success with exposure to extreme temperatures. Increased exposure to extreme temperatures in coastal areas could have population consequences for the species, especially as other studies have shown that longevity of copepods also declines at higher temperatures^56,57^.

The negative effect of warming on fitness of the tropical copepod *P*. *annandalei* supports the concern that these tropical organisms occur close to their upper thermal threshold^7,12,14^. Copepods, especially, *P*. *annandalei* are the major food source of young and small nekton species^31^. Our results suggest that with increased exposure to the extreme temperature predicted under warmer climate scenarios may yield bottom-up consequences for marine food webs by lowering the resources for higher trophic levels. Sea surface temperatures of 34 °C are often measured in the tropical coastal regions in the South East Asia^33,38,58^ and such extreme temperatures are predicted to occur more frequently and to last longer in the coming years^4,59^. The temperature-induced reduction in body size of *P*. *annandalei* we observed may also have an influence on its grazing rate as copepods (e.g. *Centropages typicus*^60^, *Acartia omorii*^61^, *A*. *tonsa*^46^) typically show a positive correlation between body size and grazing rate. The South China Sea is one of the most polluted regions on Earth^62^ and the majority of its coastal marine ecosystems are impacted by multiple stressors from anthropogenic activities (e.g. pollutions, overfishing, climate change)^63,64^. The combined effects of reduced body size and lower reproductive success of a key secondary producer under warming may be compounded by the presence of additional stressors (fisheries, pollution)^65^ and with consequences for ecosystem function and the provision of ecosystem services.

## Methods

### Study species

Adult copepods *Pseudodiaptomus annandalei* were collected from a coastal aquaculture pond in the Cam Ranh Centre for Tropical Marine Research and Aquaculture in January 2018. The mean prosome length and SE of F0 males and F0 females was 755.6 ± 2.71 and 927.1 ± 3.6 µm, respectively (n = 100 individuals per sex). The mean clutch size and SE of F0 females was 16 ± 1 eggs (n = 25). During sampling, the temperature in the pond varied between 28 and 29 °C, and the salinity was 21 ppt. The copepods were gradually acclimated to the laboratory condition at 30 and 34 °C over three days (equivalent to one-third of the generation time^66^) before introducing them to the experimental temperatures for producing nauplii to start the F1 generation. During the acclimation period, copepods were fed *ad libitum* on the haptophyte *Isochrisys galbana* (30.000–35.000 cells L^−1^ ^33^). They were kept under a photoperiod of 12L: 12D (light: dark cycle). Salinity was kept stable at 20 ppt and dissolved oxygen was 5–7 mg L^−1^.

### Experimental design

To test for the effect of warming on the fitness of the tropical copepod *Pseudodiaptomus annandalei* across three generations, we conducted a multiple generational exposure experiment in which copepods were reared at 30 and 34 °C. Each treatment had three replicates that were 5-L bottles, containing approximately 1,500 individuals (300 ind. L^−1^) similar to the copepod density observed in the pond. We chose the temperature of 30 °C as the control as it is close to the temperature measured in the field where *P*. *annandalei* is abundant^33,38,58^, and 34 °C simulated a temperature extreme that occurs frequently during the summer period.

### The cultures of F1, F2 and F3 generations

To start the F1 generation, 600 F0 females carrying two egg sacs were isolated and assigned randomly to 6 1-L bottles (100 females per bottle and three bottles per temperature), each filled with 900 ml filtered seawater. They were fed *ad libitum* on *Isochrisys galbana* (30,000–35,000 cell mL^−1^, equivalent to 800–900 µC L^−1^ ^33,67^). After incubating at 30 and 34 °C for 30 h, it was expected that 90% of eggs would hatch (Grønning, Doan, Dinh, Dinh and Nielsen, under review). We collected nauplii (approximately 1,500 individuals per 1-L bottle) to start a culture in one 5-L glass bottle (three cultures per temperature). The rearing conditions such as temperature, light regime, salinity and dissolved oxygen were similar to the acclimation period. The rearing water and algal food were renewed every two days. When F1 reached adult stage, we collected all of the adults through a filter (mesh size = 200 µm) and allowed them to produce nauplii for 30 h to start the F2 generation. Nauplii were collected, and the total number of nauplii in each bottle was determined. We used approximately 1,500 nauplii per experimental bottle to start the F2 generation. Adults were used to measure the prosomal length, the clutch size, the hatching success and the nauplii production (see details further below). The F3 generation was initiated from F2 in the same way.

To identify the development stages of *P*. *annandalei*, we daily took a subsample (100–300 ml) from one additional 5-L bottle per temperature treatment that was set up in parallel with three 5-L experimental bottles at 30 and 34 °C. Samples were filtered onto a filter (25 µm) and the content was poured on a petri dish and fixed by Lugol (4%). Developmental stages were identified based on Golez *et al*.^66^. The relative abundance (%) of nauplii, copepodites and adults in the samples was determined daily until all individuals moulted into adults.

### Response variables

Adult males and females (10–34 individuals per sex depending on the abundance) were randomly selected from each of the six 5-L bottles for measuring the size (prosome length) at maturity. The prosomal length was measured using a microscope (SZ51, Olympus, Japan). The biomass of copepods was calculated using the equation of Rayner *et al*.^68^.

1 $${\rm{C}}=2.19\times {10}^{-9}\times {{\rm{L}}}^{3.136}$$

where C = biomass in carbon unit (μg C) and L = prosomal length (µm).

Clutch size was defined as the number of eggs in the two egg sacs per female. To determine the clutch size, 9 to 13 females per experimental bottle were collected and fixed in Lugol (4%). The egg sacs were opened with a needle under a stereo-microscope (Olympus) and the number of eggs in two sacs was counted. The specific clutch size was also determined by normalizing the clutch size (0.067 µg C egg^−1^, Grønning, Doan, Dinh, Dinh and Nielsen, under review) for the biomass of the females (µg C of eggs per µg C of the female).

To investigate the effect of temperature on the hatching success of F1, F2 and F3, in each generation we collected 12 females carrying two egg sacs from each of six 5-L experimental bottle, and assigned them individually into a 12-well plate (Thermo Fisher Scientific). Each well was filled with 3 mL of clean sea water (temperatures of 30 or 34 °C, salinity = 20 ppt) and the food *I*. *galbana* at the concentration of 30,000–35,000 cell mL^−1^. Plates were placed in water baths at their corresponding temperatures. After 30 h, we checked for the hatching in each well under a stereo microscope (SZ51, Olympus, Japan). The hatching success was determined as 1 for a female with hatched eggs and 0 for a female with unhatched eggs.

### Nauplii production

We randomly collected 25 females (each carrying two egg sacs) from each temperature, and assigned them to five 125-ml glass bottles (5 females per bottle) at each temperature. They were fed on *I*. *galbana* (30,000–35,000 cell mL^−1^) for 30 h. The content was then filtered (mesh size = 25 m) and fixed in Lugol (4%). The total number of nauplii in each sample was counted using a stereo microscope (SZ51, Olympus, Japan).

### Statistical analyses

We tested for the effects of temperature, generation, and their interaction, on the size of maturity for females and males using generalised mixed models. As observations were nested within bottle, bottle was treated a as a random effect (Gaussian link, GLMMIX function). To test for the effects of the experimental treatments on the fitness parameters, we modelled the three response variables: clutch size, specific clutch size, and hatching success, as a function of the fixed effects (temperature, generation and their interaction). Observations were nested within the random effect ‘bottle’, and we used the GLMMIX function within SAS9.4. Clutch size and specific clutch size were modelled with the Poisson function, while hatching success was modelled with a binomial error structure and the logit link function. We modelled nauplii production and specific nauplii production as a function of temperature, generation and their interaction with linear models. Statistical differences were considered significant if P < 0.05. All statistical analyses were performed in SAS 9.4 (SAS Institute Inc., Cary, NC, United States). Data are presented in the figures as means + SEs.

### Data deposition

Data for this study are available at the Dryad Digital Repository when the manuscript is accepted for publication.

## Supplementary information

Supplementary Dataset 1
