# Author Correction: Extreme temperature impairs growth and productivity in a common tropical marine copepod

**DOI:** 10.1038/s41598-020-60005-6

**Published:** 2020-02-19

**Authors:** Nam X. Doan, Minh T. T. Vu, Hung Q. Pham, Mary S. Wisz, Torkel Gissel Nielsen, Khuong V. Dinh

**Affiliations:** 1 Cam Ranh Centre for Tropical Marine Research and Aquaculture, Institute of Aquaculture, Nha Trang University, No 2 Nguyen Dinh Chieu Street, Nha Trang City, Vietnam; 2 Present Address: World Maritime University, Fiskehamnsgatan 1, Malmö, Sweden; 3 National Institute of Aquatic Resources, Technical University of Denmark, Lyngby, Denmark

Correction to: *Scientific Reports* 10.1038/s41598-019-40996-7, published online 14 March 2019

This Article contains a typographical error in the Methods section under subheading ‘Nauplii production’ where,

“The content was then filtered (mesh size = 25 m) and fixed in Lugol (4%).”

should read:

“The content was then filtered (mesh size = 25 µm) and fixed in Lugol (4%).”

